# Electrodeposition of Amorphous Cobalt–Phosphorus Coating

**DOI:** 10.3390/ma18214883

**Published:** 2025-10-24

**Authors:** Noam Eliaz, Gal Weisman, Amit Kohn, George Levi, Brian A. Rosen, Alexey Moshkovich, Lev S. Rapoport

**Affiliations:** 1 Department of Materials Science and Engineering, Tel Aviv University, Ramat Aviv, Tel Aviv 6997801, Israel; galb19920@gmail.com (G.W.); akohn@tauex.tau.ac.il (A.K.); barosen@tauex.tau.ac.il (B.A.R.); 2 Center for Nanoscience and Nanotechnology, Tel Aviv University, Ramat Aviv, Tel Aviv 6997801, Israel; georgelevi@tauex.tau.ac.il; 3 Holon Institute of Technology, 52 Golomb St., P.O.B. 305, Holon 5810201, Israel; alexeym@hit.ac.il (A.M.); rapoport@hit.ac.il (L.S.R.)

**Keywords:** cobalt-phosphorous coatings, electrodeposition, amorphous films, nanocrystalline, thermal stability, adhesion, wear resistance, corrosion resistance

## Abstract

Amorphous cobalt-phosphorous (CoP) coatings are a candidate to replace hard chromium and other traditional coatings. Here, electrodeposition of both amorphous and crystalline CoP coatings was performed at room temperature and in an air environment. The bath composition and deposition conditions were optimized to offer a low cost, low maintenance, and safe process. The effects of various deposition variables such as solution composition, pH, duration, and mixing parameters were studied, and the reproducibility of the process was demonstrated. Selected coatings were then thoroughly characterized by a variety of techniques. The best amorphous/nanocrystalline coating contained ca. 6.4 wt.% P after 1.2 h of deposition, and 7.2 wt.% P after 4 h of deposition. The best crystalline coating contained ca. 2.7 wt.% P after 1.2 h of deposition and between 2.3 and 5.5 wt.% P after 4 h of deposition. The amorphous coating had excellent mechanical properties: a high hardness (7.8 ± 0.7 GPa), high Young’s modulus (153 ± 9 GPa), and surprisingly low coefficient of dry friction (between 0.11 ± 0.02 and 0.17 ± 0.01). The coating could not be scraped from the substrate using a diamond scalpel blade. In a standard adhesion test, the sample failed neither cohesively within the coating nor adhesively between the coating and the substrate. In the as-deposited conditions, the structure was uniform, nanocrystalline, or had nanocrystals embedded in an amorphous matrix. The crystallization temperature of the amorphous alloy was 284 °C, and the phase transformation occurred only between 300 and 400 °C. The coatings developed and comprehensively characterized herein may be considered for aerospace, magnetic storage, fuel cells, water splitting, and other applications.

## 1. Introduction

Most metals and alloys are crystalline, exhibiting long-range order (LRO) of atomic positions. By contrast, amorphous metal alloys (also termed metallic glasses or glassy metals) lack LRO and display only short-range order (SRO) [1,2,3]. Like oxide glasses, metallic glasses show a glass-transition temperature, *T*g. When heated above *T*g, they relax and crystallize (typically at *T*x > *T*g), losing many of their distinctive properties [2,3].

Since the 1960s, amorphous metals and alloys have been intensively studied because their topological disorder and absence of LRO confer combinations of properties that are difficult to realize in conventional polycrystalline metals, including high hardness and wear resistance (owing to the absence of dislocation-mediated plasticity), often excellent (composition-dependent) corrosion resistance, attractive luster (no grain contrast), and in some systems, useful soft-magnetic behavior and even biocompatibility [2,3]. These systems therefore continue to draw interest for structural protection, functional coatings, and magnetic and electrochemical devices.

Within this space, cobalt–phosphorus (Co–P, or CoP) coatings stand out as hard, wear-resistant layers with attractive corrosion behavior and soft-magnetic characteristics. Electrodeposited CoP shows strong wear and hardness performance suitable for protective coatings [4,5], and amorphous deposits can outperform nanocrystalline/crystalline counterparts in corrosion protection when the composition is tuned [6,7]. CoP multilayers and wires offer useful soft-magnetic and magnetoimpedance responses for sensor devices [8,9,10]. For soft-magnetic applications and sensor elements (e.g., giant magnetoimpedance, GMI), electrodeposited CoP films and multilayers offer tunable saturation and anisotropy through composition and stacking design [8,9,10], while earlier studies on magnetic anisotropy and intermediate-range order in Co–P alloys underscore the sensitivity of magnetic response to local structure [11]. For tribocorrosion environments, residual stress management, microstructure, and P content together influence wear and aqueous corrosion, and comparisons between amorphous and nanocrystalline deposits suggest potential protection advantages for amorphous CoP when composition is appropriately tuned [6,7]. In electronics manufacturing, related Co-(W)–P alloys have been explored as conformal diffusion barriers for Cu metallization, again linking performance to nanostructure and phase state [12]. In engineering practice, CoP is also considered among the more promising hard-chromium replacements, linking environmental drivers with performance targets [13].

Beyond protection and magnetics, CoP has emerged as a noble-metal-free electrocatalyst for hydrogen and oxygen evolution (HER/OER) [14,15,16]. Mechanistic and stability studies reveal that interfacial reconstruction and adsorbate-driven surface states govern alkaline HER behavior [17,18], that phase (CoP vs. Co_2_P) and support interactions matter [19], that H-adsorption energetics on low-index CoP planes correlate with activity trends [20], and that electronic-structure descriptors (e.g., unoccupied (3d) proportion) rationalize dopant effects under alkaline conditions [21]. These insights frame CoP as a tunable platform where composition, local coordination, and surface chemistry jointly determine performance [14,15,16,17,18,19,20,21].

Achieving and controlling the amorphous state in CoP is central to unlocking these property sets. Across electrodeposited studies, an amorphous structure is typically obtained once P exceeds ca. 6 wt. % (10.8 at.%) [5], with some reporting a minimum near 6.7 wt.% (12 at.%) [6,22]. Precise values and the width of the amorphous regime depend on bath chemistry and deposition conditions. Because P content also modulates hardness, corrosion behavior, and thermal stability—and because post-deposition heat treatment can relax or crystallize the deposit, with concomitant changes in properties—composition/phase control is essential when mapping processing–structure–property relations [4,5,6,22].

From an electrochemistry processing standpoint, CoP can be produced both by electroless deposition and by electrodeposition. However, the literature on electrodeposited CoP, though spanning several decades, remains comparatively sparse and fragmented relative to NiP, especially in terms of unified process windows that couple bath composition, P content, phase state, and multi-property performance in a single study. Beginning in 1946, the foundational work of Brenner, Riddell, and others established alkaline hypophosphite-based chemistries for the codeposition of P with Ni or Co and clarified the roles of the reducing agent and complexation [23,24,25,26,27].

Electrodeposition has several advantages compared to electroless plating [28,29,30]: (1) less monitoring of the solution is required throughout deposition; (2) precise control over the thickness and uniformity of the coating; (3) less expensive (due to cheaper chemicals and shorter process times); (4) increased scalability (can create higher production volumes with shorter turnaround) [13]; and (5) improved corrosion resistance. Electrodeposition also enables meniscus-confined electrochemical printing (EcP) [31,32]. These advantages are pertinent for aerospace-relevant hard coatings—where thick, conformal, low-defect films are required—and for patterned functional layers in microsystems. In parallel, the broader electrocatalysis community has codified electrodeposition as a versatile route to functional catalytic films and (hydro)oxide/oxyhydroxide phases for water splitting [33]. Alternative media (e.g., hydrophilic ionic liquids) further expand the accessible Co–P space [34].

Recent studies underscore the tunability of electrodeposited CoP across substrates, morphologies, and interfaces: one-step, stable noble-metal-free Co–P [35]; flexible electrodes via in situ CoP deposition on carbon-nanomaterial-doped PPS [36]; bath additives (e.g., saccharin) that regulate the interfacial structure and wetting to boost HER [37]; and device-level integrations such as solar-driven thermoelectrocatalysis with electrodeposited Co_2_P architectures [38] or photoelectrochemical (PEC) water oxidation wherein thin electrodeposited CoP films improve hematite anodes [39]. On porous carbon membranes, hierarchical CoP/C architectures can be built from NaH_2_PO_2_ media by electrodeposition plus some mild thermal steps [40]. Heterostructure engineering—crystalline/amorphous CoP@Ni/Fe–P [41], plasma-etched Ni foam followed by CoP/NiO electrodeposition [42], potentiostatic CoFe–P on Ni foam [43], one-step Co–P/Fe_3_O_4_ films [44], and ternary Co–P–B [45], and Co_2_P@Cu interfaces [46]—illustrates how composition and interfaces can be tailored by deposition to enhance catalytic metrics.

Despite these motivations, several gaps remain. Reported electrodeposition studies often focus on a subset of variables (e.g., current waveform or hypophosphite concentration) without systematically mapping interactions among bath composition, complexing environment, pH, agitation, and time on both P incorporation and amorphous/crystalline balance [4,25,28,47]. In addition, while individual properties—hardness, wear, magnetic behavior, or corrosion—have each been documented, fewer works provide a unified comparison of microstructure, thermal stability, mechanical/tribological performance, adhesion, and corrosion for amorphous versus crystalline CoP grown from closely related baths. Addressing these gaps would enable a more defensible selection of processing space for industrial hard-coating use (including potential replacements for hard chromium coatings) and provide transferable guidelines for CoP-based functional layers across magnetic, electronic, and catalytic devices [4,8,48].

The overarching goal of this project is to generate a consolidated processing–structure–property map for CoP coatings grown under a single, internally consistent framework. After carefully reviewing the currently available literature, the following research objectives were defined:

**(i) Process development.** Devise a simple, scalable electrodeposition route to amorphous CoP using simple bath chemistries with as few ingredients as possible, at or near room temperature (RT), without inert atmospheres, glove boxes, or templates. A benign complexing agent (glycine) was added to stabilize Co ions and promote controlled P incorporation, targeting amorphous CoP with enhanced properties.

**(ii) Process optimization.** Systematically vary bath composition, pH, deposition duration, and agitation (stirring) to define a robust, low-cost, low-maintenance, and safe process window that yields uniform coatings at practical rates; demonstrate reproducibility.

**(iii) Processing–structure–property mapping.** Characterize the microstructure, chemistry, thermal stability, hardness, Young’s modulus, wear resistance, adhesion, and corrosion resistance of selected amorphous CoP coatings in comparison to their crystalline counterparts. To this aim, scanning electron microscopy (SEM) with energy-dispersive X-ray spectroscopy (EDS), surface roughness, differential scanning calorimetry (DSC), room-temperature and high-temperature X-ray diffraction (RT-XRD (Room-Temperature X-Ray Diffraction), and HT-XRD (High-Temperature X-Ray Diffraction)), transmission electron microscopy (TEM), adhesion pull-off and tape tests, nanoindentation tests, and wear and corrosion tests were performed.

## 2. Materials and Methods

### 2.1. Materials, Chemicals, and Consumables

The working electrode (WE) substrate material for all of the experiments and characterization was copper of one of three types: (1) Cu specimen with a geometry of a rotating disk electrode (RDE). This specimen was placed in a sealed Teflon holder and had an active surface area of 2.83 cm^2^. (2) 99.9% pure Cu foil with dimensions of 5 mm × 50 mm × 0.2 mm (Alfa Aesar, Haverhill, MA, USA). (3) 99.9% pure Cu foil with dimensions of 50 mm × 10 mm × 2 mm (L × W × t) (Sofia Med SA, Sofia, Bulgaria).

The solutions used for electrodeposition contained CoSO_4_·7H_2_O powder (Cobalt (II) sulfate heptahydrate, 98%, Alfa Aesar, Lancashire, UK), NH_2_CH_2_CO_2_H powder (glycine, 99%, Alfa Aesar, Lancashire, UK), H_3_PO_3_ powder (phosphorous acid, 98+%, Alfa Aesar, Lancashire, UK), and in some cases also H_3_PO_4_ (phosphoric acid, 85%, Loba Chemie, Mumbai, India). The deposition of Co is more dominant than that of P, hence, glycine was added as a complexing agent for Co [49,50] to promote deposition of P and exceed the minimal P:Co ratio required to allow for formation of an amorphous structure.

### 2.2. Electrodeposition

Electrodeposition was carried out in a three-electrode cell. The substrate WE was the cathode of the electrochemical cell and was made of Cu as described in Section 2.1. A platinum sheet (20 mm × 20 mm) was the counter electrode (CE) and a saturated calomel electrode (SCE) was the reference electrode (REF, *E*_SCE_ = +0.241 V vs. SHE).

Substrate preparation was both mechanical and chemical. The WE was cleaned and polished before each experiment to ensure a “fresh” metallic copper surface and good wettability, thus improving both coating adhesion to the substrate and repeatability. Preparations of the Cu WE were as follows:

1. Cleaning in isopropanol (alcohol) in a sonicator (model UR 1 from Retsch GmbH, Haan, Germany, operation frequency 35 kHz, volume 5.7 L) for 15 min.
2. Polishing on aluminum oxide flexible foams (3M, Saint Paul, MN, USA): “fine” (grit 320 to 400), “superfine” (grit 500 to 600), and “microfine” (grit 1200 to 1500).
3. Cleaning in isopropanol in a sonicator for 15 min.
4. Chemical polishing in a concentrated acid solution consisting of H_3_PO_4_, HNO_3_, and CH_3_COOH (1:1:1 vol. ratio).
5. Cleaning with deionized (DI) water.
6. Drying with a cold-air fan.

To prepare 100 mL of solution No. 0, 7.0275 g of CoSO_4_·7H_2_O powder was weighed and added to 90 mL of DI water in a flask and dissolved by magnetic stirring. Then, 2.2521 g glycine powder was weighed, added to the flask, and dissolved. Next, 0.999 g H_3_PO_3_ powder was weighed, added to the flask, and dissolved. Then, 0.53138 mL H_3_PO_4_ was added using a micropipette and dissolved. NaOH diluted in DI water (1:5 vol. ratio) was added using a pipette in order to achieve the desirable pH. The pH was measured using a pH 510 pH/mV/Temperature or Ion 510 Ion/pH/mV/Temperature bench meter (Eutech Instruments Pte Ltd., Singapore). Then, DI water was added until a final volume of 100 mL was reached, at which point the reported pH was measured. The preparation of solution No. 1 was the same, but excluded H_3_PO_4_. Solutions Nos. 2 and 3 were similar to solution No. 1, except for the content of glycine (4.5042 g of glycine powder) and the pH (Solution No. 3). Solution No. 4 was similar to Solution No. 3, except the content of CoSO_4_·7H_2_O powder was 2.811 g. The accuracy of the added components was 0.001 g. Thus, six different solutions were prepared, as listed in Table 1.

Initially, cyclic voltammetry (CV) experiments were conducted for all of the solutions in Table 1 in order to determine the deposition potential range. These scans were conducted at RT at a sweep rate of 35 mV/s, from positive to negative potential (forward scan), and then from negative to positive potential (reverse/backward scan). Based on these experiments, the rest of the work was done by potentiostatic deposition at −0.8 V vs. SCE. All experiments were carried out at RT in an air environment for either 1.2 or 4 h. We first used RDE WEs and an RDE Modulated Speed Rotator (MSR) (Pine Research Instrumentation, Durham, NC, USA) for electrodeposition. From these experiments, we observed that **amorphous coatings were obtained from stirred solutions, whereas crystalline coatings were obtained from unstirred solutions**. We then used Cu foils and a BioLab potentiostat with EC-Lab v.11.36 software (BioLogic, Seyssinet-Pariset, France) for the remainder of the electrodeposition experiments (mostly with magnetic stirring).

### 2.3. Coating Characterization

SEM and EDS were used to characterize the surface morphology of the coatings and elemental mapping, respectively. SEM images were acquired in a Quanta 200F FEG (FEI, Waltham, MA, USA) microscope operated in the high-vacuum mode, with an accelerating voltage of 10–20 kV. The chemical composition of the coatings was determined by EDS (INCA detector, Oxford Instruments, Abington, UK) with a 10 mm working distance (WD) from the detector and an accelerating voltage of 20 kV. Peak energies (position) on the EDS spectrum were predicted using Moseley’s law. For major elements, it was possible to obtain a precision (defined as 2σ) of better than ±1% (relative), but the overall analytical accuracy is commonly nearer ±2% owing to factors such as uncertainties in the compositions of the standards and errors in the various corrections which need to be applied to the raw data [51].

Metallographic cross-sections were prepared from selected coated samples to measure the coating thickness, characterize the interface between the substrate and the coating, and for nanoindentation measurements (to cross-validate measurements where the indentation was done on the top surface). The coated samples were placed in a custom-designed specimen holder to which EpoFix resin (Struers A/S, Rødovre, Denmark) was added. After curing, mechanical grinding, and polishing was carried out using the Tegramin-25 automatic preparation system (Struers, Cleveland, OH, USA).

Confocal scanning laser microscopy (CSLM) measurements were performed in order to analyze the surface roughness of the coated samples. The surface roughness of a material directly affects its wear resistance—high roughness will usually lower the wear resistance, as will be reflected by a higher friction coefficient. Roughness measurements were conducted using a LEXT OLS4100 laser scanning microscope (Olympus, Tokyo, Japan). This microscope has a lateral resolution of 0.12 µm and a height resolution of 10 nm height. The microscope used a 405 nm semiconductor laser as light source, photomultiplier detector, and dedicated objective lenses.

Both a tape test (according to ASTM D3359, method B [52]) and a pull-off test were employed to analyze the adhesion of the coating to the substrate. For the tape test, a manual Elcometer 107 Cross Hatch Adhesion Tester (Elcometer, Manchester, UK) was used with six teeth with a 1 mm pitch cutter blade (SH 38271) and ASTM D3359 adhesive tape (Elcometer, Manchester, UK). For the pull-off test, a PosiTest^®^ AT-A portable Automatic Pull-Off Adhesion Tester (DeFelsko, Ogdensburg, NY, USA) was used. This instrument measures the force required to pull a specified test diameter of coating away from its substrate using hydraulic pressure. The pressure is displayed on a digital LCD and represents the coating’s strength of adhesion to the substrate. In accordance with ASTM D4541 [53], ASTM D7234 [54], ISO 4624 [55], and others, the instrument evaluates the adhesion (pull-off strength) of a coating by determining the greatest tensile pull-off force that it can bear before detaching. Breaking points, demonstrated by fractured surfaces, occur along the weakest plane within the system consisting of the dolly (loading fixture, stub), glue, coating layers, and substrate. Aluminum dollies, 10 mm in diameter, were adhered to the sample with DP-420 Scotch-Weld Epoxy adhesive (3M, Saint Paul, Minnesota, MN, USA). The maximum pull-off pressure for this dolly size was 96 MPa. The pulling rate was 0.7 MPa/s. The device calculates the strength according to the following equation:

(1) $$\sigma =\frac{4F}{\pi d^{2}}$$

where *σ* is the tensile strength (MPa), *F* is maximum force applied on the area (N), and *d* is the diameter of the dolly (mm).

Dry friction tests were conducted in order to study the coefficient of friction for the CoP coating (on the Cu substrate). All of the samples for tribological tests were deposited from Solution No. 1 (see Table 1) for 4 h with stirring. The measurements of friction and wear were carried out according to ASTM G133 [56]; reciprocating ball-on-flat motion with a sliding velocity of 0.4 mm/s during 100 cycles was performed. A ball with a diameter of 2 mm made of AISI 50100 steel rubbed against the flat CoP coatings. The load of 50 g provided an initial Hertz pressure of ~1 GPa. The length of wear track was 2 mm.

Nanoindentation measurements were carried out to determine the hardness and Young’s modulus of the coated alloy. Preliminary Vickers microhardness tests with a load of 10 g failed because the indent was too large and penetrated through the thin coating to the Cu substrate. Therefore, nanoindentation tests were essential. The instrumented nanoindentation tests were conducted at RT and ambient pressure using a KLA Tencor Nano Indenter XP (KLA, Milpitas, CA, USA) with a standard Berkovich indenter. The Continuous Stiffness Measurement (CSM) mode was used, yielding modulus and hardness values continuously as a function of penetration depth. Initially, tests were conducted both on the top of the coating and in the cross-sections of CoP-coated 0.2 mm thick Cu. However, due to curling of the thin Cu foil, it was decided to prepare new samples on 2 mm thick Cu and conduct the measurements only on the top surface. The results reported herein are average values for 185 to 350 nm depth into coatings on the thicker Cu foil. Measurements were carried out at a constant strain rate of 0.05 1/s. The deposition time for these experiments was 4 h, yielding CoP coatings with a thickness of ~4.2 μm.

Corrosion tests were performed in order to characterize the corrosion resistance of the CoP coatings. Open-circuit potential (OCP) tests followed by potentiodynamic (PD) polarization tests were conducted in an aerated solution of 0.1 M NaCl at RT and a scan rate of 25 mV/s. The PD polarization tests were scanned from −1.0 V to +2.0 V. The surface area exposed to the solution was 0.567 cm^2^.

DSC was carried out to support the existence of an amorphous structure of the as-deposited coating as well as to determine the crystallization temperature and possible other phase transitions. The DSC measurements were conducted using a DSC Q20 system (TA instruments, New Castle, DE, USA) with a nitrogen gas flow of 50 mL/min and heating rates of either 10 or 20 °C/min. Some tests were run from 15 to 594 °C, others from 20 to 317 °C. Due to the exceptionally strong adhesion of the coating to the substrate, coated samples were eventually used for these tests, despite the original plan to generate powder by scraping coated samples. To obtain at least 2 mg of powder from samples coated with 2 μm thick CoP, taking into account ρ_Co_ = 8.86 g/cm^3^ and neglecting P, the total surface area of the coated samples would have to be 1.2 cm^2^. For comparison, each RDE electrode yielded only ~0.2 mg of coating.

RT-XRD was first used to verify the amorphous structure of the CoP coating. Coated “bulk” samples were used for this purpose. HT-XRD allows the study of dynamic processes and phase transformations. Here, it was used to determine the crystallization temperatures and phase transformations at temperatures as high as 400 °C. For these experiments, coated “bulk” samples were used. Originally, it was planned to use a coating powder scraped from coated samples. To obtain at least 400 mg powder from samples coated with 2 μm thick CoP, taking into account ρ_Co_ = 8.86 g/cm^3^ (and neglecting P), the total surface area of the coated samples was supposed to be 226 cm^2^ (i.e., a large coated sample of ca. 15 cm × 15 cm had to be prepared). However, due to the exceptionally strong adhesion of the coating to the substrate, scraping powder was not possible, and coated “bulk” samples had to be used. A D8 ADVANCE diffractometer (Bruker AXS, Madison, WI, USA) with a Cu anode (λ = 1.54060 Å) was used in the Bragg–Brentano geometry for both RT- and HT-XRD tests. The scan was within the range of 2θ = 10–100°, the step size was 0.02°, and the time per step was 1 s (as there are 192 channels in the detector, the real time was 192 s per step). In the case of HT-XRD, the sample was placed inside an Anton Parr XRK-900 high-temperature cell (Anton Paar, Graz, Austria) and was purged with pure nitrogen for 1 h prior to the measurement. XRD diffractograms were collected at 25, 100, 150, 200, 250, 300, 350, and 400 °C under nitrogen flow in order to monitor the crystallization of the originally amorphous CoP sample. The heating rate between temperature steps was fixed at 10 °C/min. The X-ray source was fitted with a 0.6 mm slit (equatorial) and a 2.5-degree Soller slit. The linear position sensitive X-ray detector (PSD) was also fitted with a 2.5-degree Soller slit and was open full to an angle of 3 degrees. Phase identification was done using the ICDD 2022 database in conjunction with TOPAS fitting software. The final phase identification was assigned on the basis of the evolution of the peaks with respect to temperature, the position of the peak center as compared to the values in the ICDD database, and the weighed profile R-factor (Rwp) acquired after attempting to refine the fit of the XRD diffractogram in TOPAS with the various candidate phases.

TEM was employed to characterize the structure of the CoP coatings and their interface with the Cu substrate. Both a JEM-2010F UHR (JEOL, Tokyo, Japan) TEM and a double spherical aberration (Cs) corrected Scanning TEM (STEM) Titan Themis G^2^ 60-300 (Thermo Fisher Scientific, Hillsboro, OR, USA) microscopes were used. Both microscopes were operated at an accelerating voltage of 200 kV.

The JEM-2010F microscope was applied in TEM mode for bright-field (BF) and dark-field (DF) imaging as well as parallel illumination microdiffraction. Furthermore, energy-filtered TEM (Tridiem Gatan Imaging Filter, Gatan, Pleasanton, CA, USA) was used to estimate the specimen thicknesses at around 100 nm.

The Titan Themis G^2^ 60-300 microscope was operated in STEM mode, using a convergence angle of 19.5 mrad to achieve a spatial resolution of approximately 1 Å. Imaging was obtained using a BF detector up to 64 mrad, and high-angle annular detector (HAADF), Z-contrast, at an angular range of 116 to 200 mrad. EDS mapping on this microscope was done using a Dual-X detector (Bruker, MA, USA) and analyzed in the Velox™ software package. Quantification and mapping of the composition from the EDS spectra was achieved by background correction with a multi-polynomial model of a parabolic order. An absorption correction was applied for the estimated sample thickness and a density of approximately 8.9 g/cm^3^.

A focused ion beam (FIB) was used to prepare TEM lamellas. A Helios Nano Lab 460F1 dual beam FIB (FEI, Hillsboro, OR, USA) was used. After depositing carbon and platinum protective layers, Ga ions were used for milling, employing an initial ion voltage of 30 kV and a final ion milling voltage of 5 kV. The extracted lamellas were then attached to molybdenum grids. Since the microstructure of the CoP layer was expected to be somewhere between fully amorphous and nanoscale crystals embedded in an amorphous matrix, we were concerned that high-energy Ga milling would introduce artifacts. A pre-examination of Ga ion milling at 30 kV and 5 kV indicated that the studied TEM samples represented the structure of the material.

## 3. Results and Discussion

### 3.1. Coating Electrochemistry and Deposition Potential

**Figure 1:**
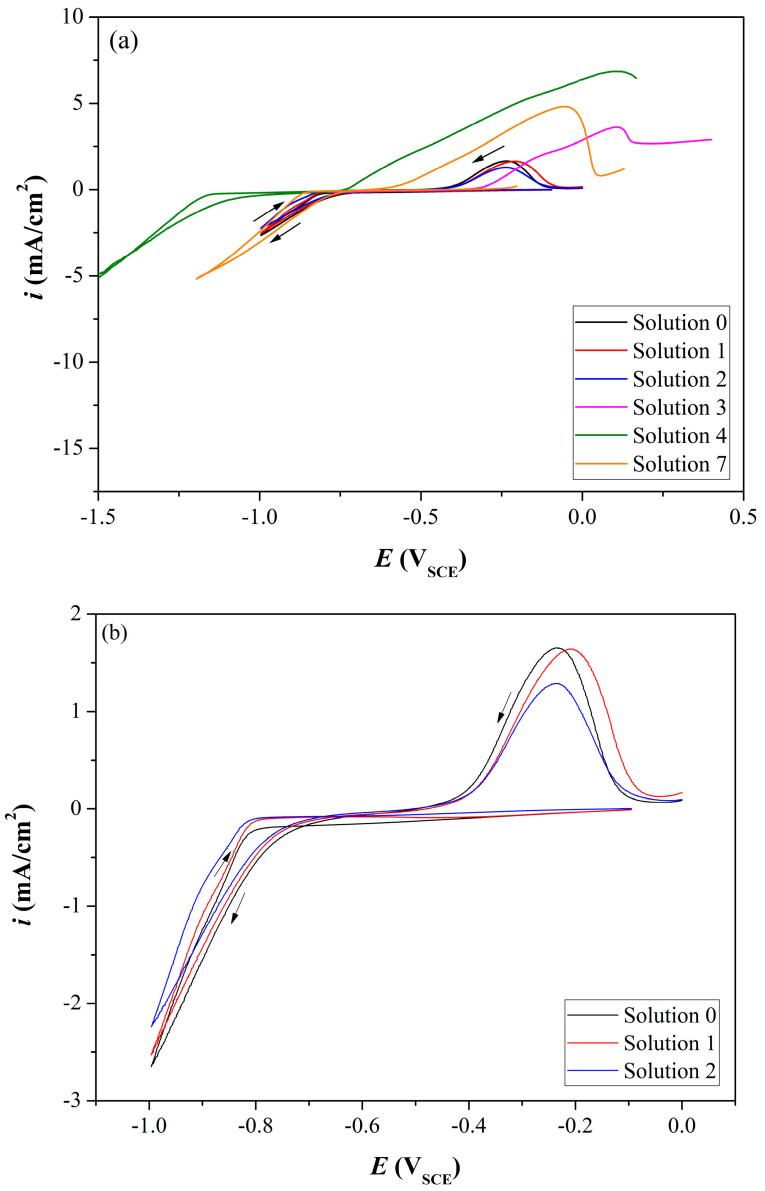
(**a**) Cyclic voltammograms for Cu electrode in six solutions. (**b**) Zoom-in for solution Nos. 0–2. The arrows show the scan direction, from positive to negative potential in the forward scan, and from negative to positive in the reverse scan.

CV experiments were first run in order to determine the CoP reduction potential from each of the six solutions in Table 1. As shown in Figure 1, the reduction of CoP started at ca. −0.8 V vs. SCE in solutions No. 0–3 and 7, and at ca. −1.14 V in solution No. 4. These values are similar to those reported before for electrodeposition of CoP [34,57]. Based on subsequent deposition tests at −0.8 V (solutions No. 0–3), −1.1, −1.2, and −1.5 V_SCE_ (solutions No. 4 and 7) for 1.2 h and complementary SEM-EDS analyses, it was concluded that **deposition at −0.8 V_SCE_ and solution No. 1 yielded the best coating**. Therefore, this combination was used for the remainder of the study. No coating was deposited from solution No. 3. Solution No. 4 was unstable and yielded a pure Co coating. Hence, no results from these two solutions are shown in the following sections. With respect to the oxidation peak in the CV (which can be ascribed to the reaction Co ⟶ Co^2+^ + 2e^−^ [34,57]), it started at ca. −0.46 V and ended at ca. −0.05 V, with the peaks positioned at −0.21 V for solution No. 1 and at −0.23 V for Solution Nos. 0 and 2 (see Figure 1b). Therefore, we can expect the coating to be fully removed, exposing the Cu substrate to corrosion, when running PD polarization corrosion tests at potentials higher than ca. −0.21 V using the same scan rate.

### 3.2. Coating’s Surface Morphology, Surface Roughness, and Thickness

**Figure 2:**
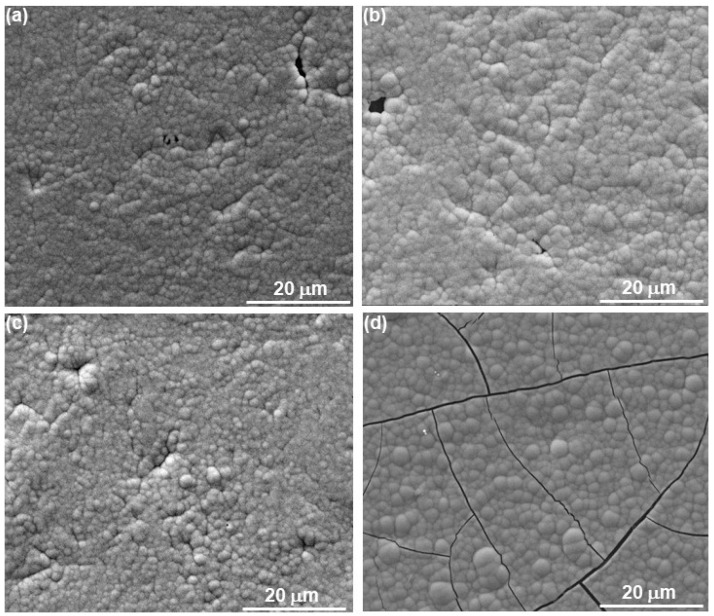
Top view SEM SE images of CoP layers deposited on Cu for 1.2 h from solutions No. 0 (**a**), 1 (**b**), 2 (**c**), and 7 (**d**), with stirring.

The surfaces of coatings deposited under stirring from Solution Nos. 0, 1, 2, and 7 were characterized by SEM (see Figure 2) using secondary electrons (SEs). A mesoscale colony surface structure (also known as a nodular or cauliflower structure) was evident. This morphology is typical of electroless/electrodeposited metals and alloys with amorphous and nanocrystalline structures, including CoP alloys [4,5,6,14,47,57,58,59,60,61,62,63]. The appearance of these 3D islands may indicate the Volmer–Weber growth mode, which is preferred in electrodeposition when the binding energy between adsorbed deposited ions is greater than the binding energy between an adsorbed ion and a substrate atom [64]. Such an appearance has been widely ascribed to a 3D instantaneous nucleation mechanism.

The addition of glycine as a complexing agent in Ni electrodeposition from an acidic bath has been found to refine the grains and increase the non-crystallinity and brightness of the coating [65]; glycine may have a similar effect in the present study. It has been suggested that the electrodeposition mechanism of CoP involves the transfer of neutral H_3_PO_3_ molecules from the bulk solution to the cathode surface, their adsorption on the cathode surface, and the reduction of the adsorbed H_3_PO_3_ to phosphorus alongside metallic Co deposition [66]. The mechanism of induced codeposition was thus explained by a concept of adsorption equilibrium of H_3_PO_3_ molecules. Finally, it should be noted that amorphous morphology is typically smoother and results in a brighter coating than nanocrystalline morphology.

In Figure 2d, “mud-cracks” were evident at the surface of the coating deposited from solution No. 7. In general, such cracks may be related to the decomposition of unstable hydrides during or soon after plating, residual stresses due to codeposition of hydrogen, defects incorporated in the coating, epitaxial growth, and a significant difference between the interatomic spacing of the substrate and deposit materials [64]. Mud-crack formation in amorphous CoP electrodeposits typically arises from a combination of internal tensile stress, volumetric shrinkage, and mechanical brittleness—characteristic features of amorphous transition metal phosphide films. The incorporation of phosphorus can distort the cobalt lattice, particularly in the amorphous phase, while hydrogen evolution during deposition introduces porosity and localized stress sites. These factors collectively contribute to significant internal stresses, which are exacerbated in amorphous materials due to their lack of LRO, limiting their ability to accommodate strain. Following electrodeposition, if the film undergoes drying (e.g., air drying, vacuum drying) or post-deposition annealing, the release of residual solvents or byproducts (such as water or hydrogen) can cause additional shrinkage. However, because the film is strongly adhered to a rigid substrate, it cannot contract freely, leading to the build-up of tensile stress. Once this stress exceeds the fracture toughness of the material, cracking occurs. The inherently brittle nature of amorphous CoP, especially in thicker films, further contributes to this behavior. Unlike crystalline metals, which can relieve stress through dislocation-mediated plasticity, amorphous CoP lacks such mechanisms, making it prone to brittle fracture. This results in a characteristic mud-crack morphology, a hallmark of tensile failure in brittle films. Thicker CoP films are particularly susceptible to cracking due to: (1) greater accumulation of internal stress over time; and (2) reduced ability to dissipate stress through the film’s thickness. In contrast, thinner films are often able to remain intact or exhibit only fine cracking. Mud-crack formation can be mitigated—or in some cases, entirely avoided—through several strategies: (1) reducing film thickness; (2) using lower current densities to enable slower, more uniform growth; (3) optimizing the bath composition to minimize hydrogen evolution; (4) incorporating additives such as surfactants or stress-relieving agents; and (5) applying controlled post-deposition annealing to gradually relieve internal stresses.

**Figure 3:**
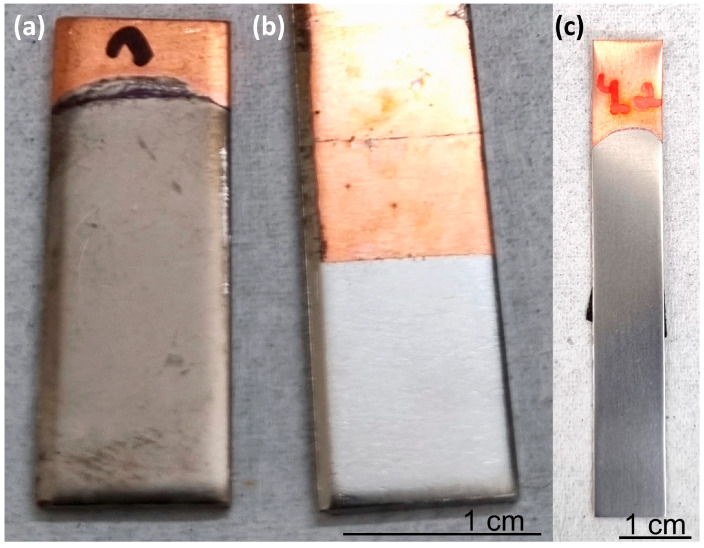
Macroscopic appearance of coatings electrodeposited from solution No. 1, with (**b**,**c**) and without (**a**) stirring.

Electrodeposition from solution No. 1 was carried out both with stirring (on a magnetic stirring plate) and without stirring. Visually, the two coatings looked different. When stirring was implemented, the coating appeared glossy (typical of amorphous alloys that lack grain structure), whereas without stirring, the coating appeared matte (which is more typical of crystalline alloys as shown in Figure 3). The difference in macroscopic appearance arises primarily from how light interacts with the surface structure at the microscopic level. Amorphous coatings have a more uniform and smooth microstructure because they lack grain boundaries and crystalline defects. This smoothness leads to specular (mirror-like) reflection of light, which gives the surface a glossy or shiny appearance. Crystalline coatings, on the other hand, are composed of many small grains with varying orientations and may have microscopic roughness at the grain boundaries. This causes diffuse scattering of light in multiple directions, making the surface appear matte or dull.

**Figure 4:**
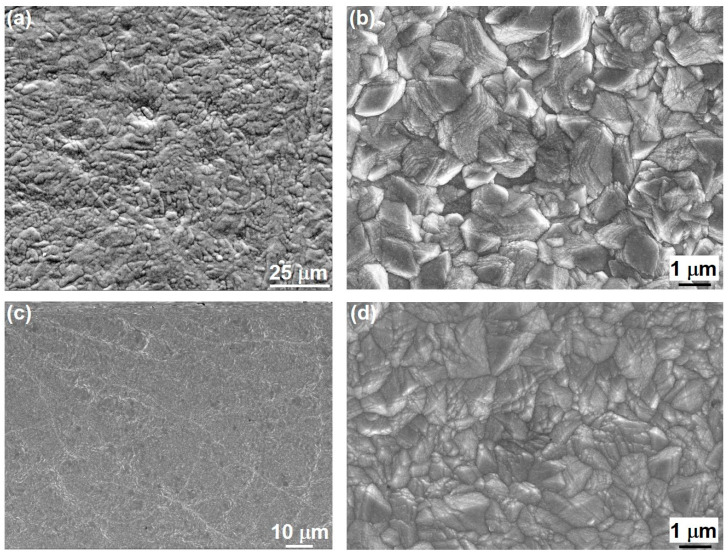
SEM SE images of electrodeposition from solution No. 1 without stirring for (**a**,**b**) 1.2 h, and (**c**,**d**) 4 h.

Both coatings appeared uniform, dense, and with good surface coverage. SEM images using SEs (Figure 4) revealed the crystalline (grains) structure at the surface of the coating deposited without stirring. This surface morphology was different than that of the amorphous coatings in Figure 2.

**Figure 5:**
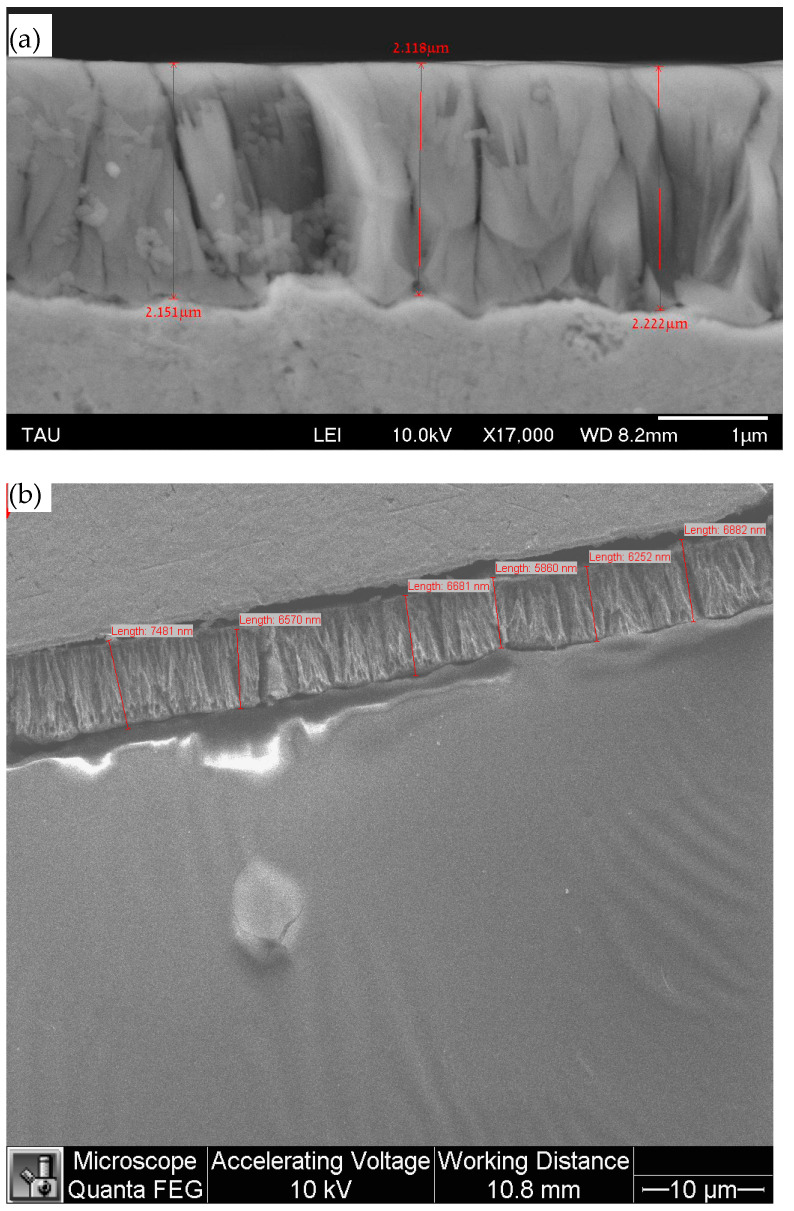
Thickness measurements on SEM SE images of the cross-sections of coatings deposited from solution No. 1 for (**a**) 1.2 h, and (**b**) 4 h. Cr coating was deposited on the cross-section in (**a**) to minimize charging and improve the image quality.

CSLM was used to measure the surface roughness of both amorphous and crystalline CoP coatings. Table 2 summarizes the average roughness, *R*a, values for the three surfaces. The values were similar for the three surfaces, although the crystalline coating was slightly rougher. It should be noted that the surface of the uncoated Cu sample was prepared mechanically prior to electrodeposition. Therefore, it may be concluded that the surface morphology of the CoP coating conforms to the asperities on the surface of the Cu substrate. This conclusion is further supported by SEM SE images of the cross-section (see, for example, Figure 5). This finding could be one of the reasons for the strong adhesion between the coating and the substrate, as found in this study (“mechanical interlocking”). The *R*a values in Table 2 match ISO grade number N2.

Coating thickness measurements on cross-sections (Figure 5) gave a thickness of 2.14 ± 0.07 μm (*n* = 6, number of measurements) for 1.2 h deposition, implying a deposition rate of 1.78 μm/h. After 4 h of deposition, the thickness of the coating was 6.62 ± 0.55 μm (*n* = 6), implying a deposition rate of 1.66 μm/h, i.e., only slightly slower. These results imply that the deposition reaction is approximately Faradaic.

### 3.3. Chemical Composition of the Coatings

**Figure 6:**
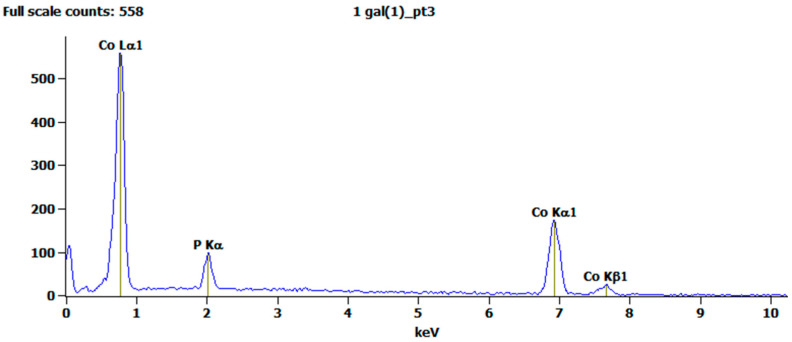
A representative EDS spectrum for a coating deposited from solution No. 1 with stirring.

The chemical composition of the coatings was determined by EDS. A representative spectrum (from a coating deposited from solution No. 1, with stirring) is shown in Figure 6. Only Co and P are evident, indicating that the coating is pure and is thick enough to prevent the appearance of the Cu substrate in the spectrum. The stability of the process and the reproducibility of results were verified.

Table 3 summarizes the typical chemical compositions of coatings deposited from different solutions and/or over different periods of time. The cracked CoP coating deposited from solution No. 7 had a significantly lower P content. Coatings deposited from solutions No. 1 and 2 had essentially the same chemical composition, except that the coating from solution No. 2 contained twice the concentration of glycine compared to solution No. 1. From Table 3, it is evident that the (nano)crystalline coatings deposited from solution No. 1 contained less phosphorous than the amorphous (combined with nanocrystalline) coatings deposited from the same solution (either after 1.2- or 4-h depositions). Yet, coatings deposited from solutions No. 0 and 7 had a similar nodular morphology in the SEM images despite containing less P. When no stirring was used, O and C contamination was found in CoP coatings deposited over both 1.2 and 4 h.

### 3.4. Corrosion Resistance of the Coatings

**Figure 7:**
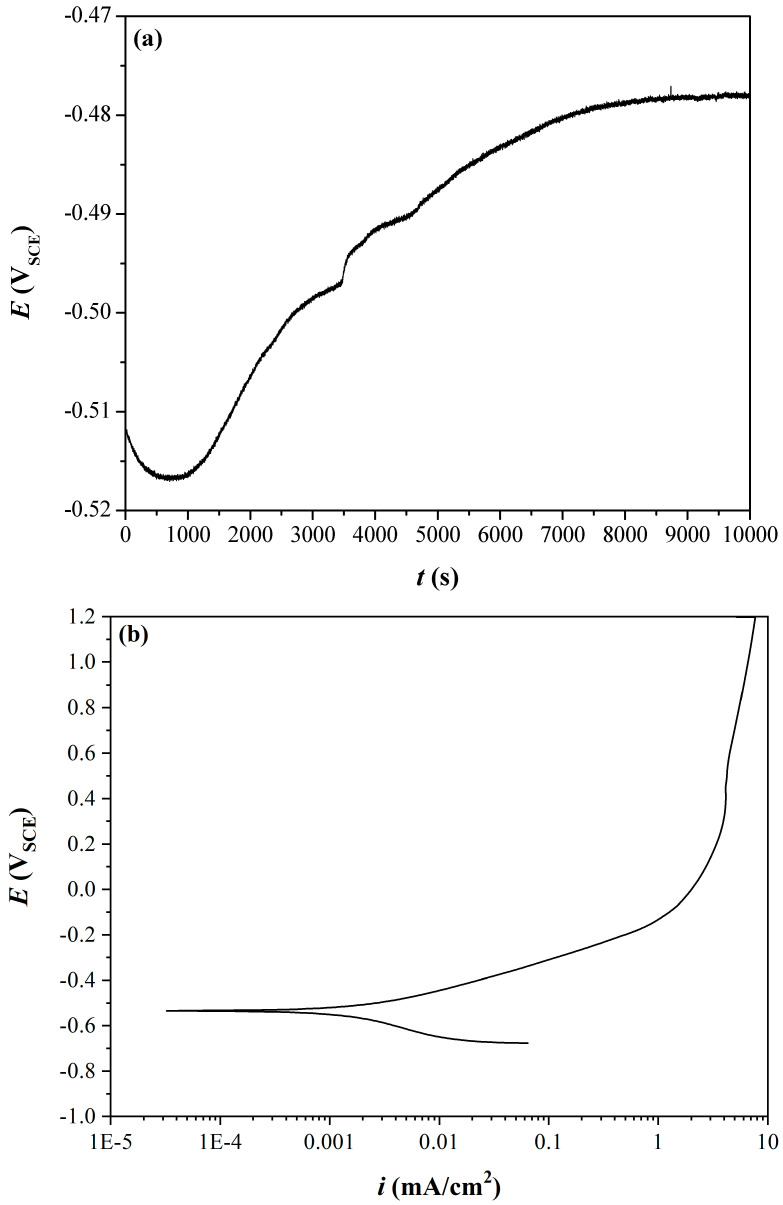
(**a**) OCP curve of CoP coating on Cu in 0.1 M NaCl at RT. (**b**) Potentiodynamic polarization curve of Cu coated with CoP from stirred solution No. 1 in 0.1 M NaCl at RT.

OCP tests followed by PD polarization tests were performed at RT in a solution of 0.1 M NaCl. The potential in the PD polarization test was scanned from −1.0 V to +2.0 V, therefore at least up to −0.46 V the curves are supposed to represent the CoP coating and not the Cu substrate (if the coating were not porous and no delamination occurred). At the end of the PD test, the Cu substrate was apparent within the circular area exposed to the solution. This could be expected, as the PD test was run well above −0.21 V. The OCP vs. time curve is shown in Figure 7a. The OCP first slightly decreased and then increased to a steady state at ca. −0.48 V_SCE_ after ca. 148 min. The stable OCP indicates that the various corrosion reactions have assumed a constant rate. The PD polarization curve is shown in Figure 7b. While a passivation regime is evident, at which *i*_pas_ = 7.6 mA/cm^2^, transpassivity is not evident up to +1.2 V vs. SCE.

Tafel extrapolation of the anodic and cathodic curves yielded a corrosion potential, *E*_corr_, of −0.54 V_SCE_ and corrosion current density, *i*_corr_, of 1.78 μA/cm^2^. The anodic and cathodic Tafel slopes were *b*_a_ = +132 mV/decade and *b*_c_ = −129 mV/decade. The corrosion potential, corrosion current density, and Tafel slopes measured in this work are comparable to those reported elsewhere when considering similar P content and structure of the CoP phase [4,6,7,60,62,67,68].

Li et al. [4] reported that as the P content was increased, *E*_corr_ shifted in the positive direction and *i*_corr_ decreased. It was suggested that phosphorous reacts with water to form a layer of adsorbed hypophosphite anions on the surface of the coating, which in turn prevents the water from reaching the electrode surface, thereby preventing further dissolution of cobalt. 

In corrosion testing, the higher *E*_corr_ and the lower *j*_corr_ are, the better the corrosion performance of the material is [30]. Kosta et al. [60] studied the corrosion behavior of nanocrystalline Co-(1–3 wt.%)P in an aerated 3.5% NaCl solution at RT. The measured *i*_corr_ was 0.25 μA/cm^2^ for the as-deposited sample, the *E*_corr_ was −0.503 V_SCE_. Kosta et al. [67] further compared the corrosion properties of similar nanocrystalline coatings under the same conditions to those of hard chromium coating. The measured values were *E*_corr_ = −0.503 V_SCE_ and −0.545 V_SCE_, respectively, whereas the *i*_corr_ values were 0.25 and 0.40 μA/cm^2^, respectively. Ezhilselvi et al. [62] observed a significant increase in P content on the surface of coatings with an initially lower P content, reflecting active dissolution of these coatings in the corrosion medium, whereas coatings initially richer in P showed only a marginal increase in P after corrosion and better corrosion resistance.

Regarding the Cu substrate, its corrosion behavior under similar conditions is already well documented in the literature and was therefore not investigated further in this work. It suffices to note that the following values were reported [69] for pure Cu in aerated 0.1 M NaCl at 25 °C: OCP ≈ −0.225 V_SCE_, *E*_corr_ = −0.195 V_SCE_, and *i*_corr_ = 5.16 μA/cm^2^. It is well established for Cu in chloride media that, in a stirred cell, *i*_corr_ increases relative to the quiescent value and *E*_corr_ shifts only modestly (with the direction depending on the condition of the surface films), because stirring enhances O_2_ mass transport [70].

### 3.5. Hardness, Young’s Modulus, Adhesion, and Friction Properties, of the CoP Coating

Tape tests were carried out using samples made of amorphous CoP coatings on Cu substrates to measure the adhesion. A classification of 5B, i.e., the highest rating, was found. Zero percent of the area was removed, and the coating remained completely on the substrate. Qualitatively, it can be claimed that the adhesion between the coating and the Cu substrate is excellent. Exceptionally strong adhesion between Co and Cu has been reported before, albeit without a physical explanation [71].

When one observes strong adhesion at an alloy coating/metal substrate interface in electrodeposition, the following mechanisms or factors are often invoked (and likely act in concert): **(1) Interfacial bonding.** Co–Cu bonding is plausible. The Co in the CoP deposit may form metallic bonds with the Cu substrate atoms at the interface, especially if the interface is clean of oxides. Co may reduce right at the Cu surface, leading to some intermixing or interpenetration. Even a very thin interdiffused zone will reduce the abruptness of the interface and aid adhesion. The presence of P adds a non-metallic character; P might bond or hybridize with substrate atoms, making additional chemical interactions at the interface. Some studies on metal-phosphide/substrate interfaces note shifts in bonding energies, indicative of interfacial coupling, which can strengthen bonding. The interfacial coupling might also lower the binding energy of P or shift orbital energies, contributing to stronger bonding across the interface [72]. In designing cobalt phosphide coatings, some authors deliberately tailor interfacial wetting or bonding structures to enhance interface coupling [37]. **(2) Mechanical interlocking, surface roughness, and anchoring.** Displacement reactions or local dissolution–reprecipitation in the early stages can roughen or etch slightly, increasing mechanical interlocking [73]. In [37], the authors deliberately adjusted deposition conditions so that the interface was favorable (wettability, morphology, coupling) to improve performance. That suggests that interfacial engineering is possible in CoP systems. **(3) Pretreatment/substrate cleaning.** Often, achieving high adhesion requires that the copper substrate be well-prepared (oxide removal, polishing, pretreatment) so that fresh copper atoms are exposed and can bond with the deposit. **(4) Avoidance of defects such as voids:** Optimizing deposition parameters (current density, bath composition, agitation, and bath additives) to achieve dense, uniform deposition is key to minimizing interfacial defects. **(5) Stress state.** If the CoP deposit has internally compressive stress (not excessive), it may “press” the film onto the substrate, enhancing contact. **(6) Fine grain structure.** A fine structure helps reduce mismatch stress and improves uniform adhesion. For instance, ultra-fine equiaxed grains formed in CoP coatings were claimed to contribute to desirable mechanical properties [60]. Lu et al. [74] used nanoscratch testing and TEM to correlate adhesion with CoP microstructure. They reported that nCoP adheres significantly better than amorphous Co–P on Cu. Crucially, they observed an interfacial Co–Cu phase (formed when the Co–P is nanocrystalline) and attributed the higher adhesion to this intermetallic Co–Cu layer acting as a chemically bonded transition zone at the interface.

In pull-off tests, a sample made of amorphous CoP coating on Cu was adhered with DP-420 adhesive to an Al plate to prevent distortion of Cu during the test. No adhesive failure between the CoP coating and the Cu substrate was observed. Instead, the failures were either between the Cu and the Al plate or between the dolly and the coating. The DP-420 adhesive remained completely on each dolly, with no coating on the dolly thanks to the strong bonding between the coating and the Cu substrate. In both cases, the normal stress was about 14 MPa. The adhesion between the CoP coating and the Cu substrate must therefore be higher. This test is supported by the fact that it was not possible to remove the CoP coating from the substrate for DSC tests.

The friction properties of the amorphous CoP coating on the substrate were determined by dry friction (tribological) tests. Before testing, all of the samples were characterized by SEM-EDS to be sure that both the surface morphology and the chemical composition were the typical ones and matched those reported above. Differences in either chemical composition or surface morphology could be responsible for variation in wear resistance. Table 4 summarizes the wear test results. The effective coefficient of friction of all the samples was 0.14 ± 0.02. This value was lower compared to those reported elsewhere (where higher values of 0.33, 0.38 and 0.48 were reported for amorphous structures with a similar Co:P ratio) [4,5]. Barzegar et al. [5] reported coefficient of friction values between 0.60 and 0.71 for amorphous coatings with different P contents. After thermal treatment, the values reduced to between 0.31 and 0.55. Kosta et al. [67] reported a friction coefficient of 0.55–0.60 in nanocrystalline CoP (nCoP) when using an alumina ball, and 0.60–0.65 when using a WC/Co ball. As the coefficient of friction is lowered (and the hardness is higher), the wear resistance increases. McCrea et al. [75] reported a coefficient of friction of 0.4–0.6 and hardness of 530–600 VHN for nCoP. Improved friction properties of studied coatings can be explained by the large amount of amorphous phase.

The hardness of the material affects its wear resistance. The elastic modulus is another important mechanical property. To determine both locally, nanoindentation tests were carried out. Both the surface morphology and the chemical composition of the tested samples were characterized. As reported before, the surface of the crystalline sample contained microcracks and a globular grain shape, whereas the surface of the amorphous sample was smoother and free of microcracks. The EDS results also reproduced previous results, showing a higher P content in the amorphous alloy (Table 5).

As evident from Table 6, the hardness of the amorphous coating was 1.5 times higher than that of the crystalline coating. The average elastic modulus of the crystalline CoP coatings is higher than that of the amorphous CoP coating. The higher roughness of the crystalline coating (see Table 2) may lead to a very large dispersion in the elastic modulus because, for calculation of the modulus, it is presumed that the sample is smooth and isotropic. This uncertainty is not critical for the hardness results, since the mean values were quite different compared to the standard deviation. The hardness values reported herein were similar to or higher than the values reported elsewhere [4,5,58,59,60,76].

### 3.6. Microstructure of CoP Coatings

The electrodeposition of Co–P typically proceeds via Faradaic charge transfer under applied potential or current. Key aspects include: **(1) Co and P precursor reduction**. The bath contains cobalt salts, often complexed to moderate reduction kinetics, plus a phosphorus source (e.g., hypophosphite, phosphorous acid, phosphates, phosphonates, or phosphate ions in special form). At an applied cathodic potential, cobalt ions are reduced to metallic Co. **(2) Codeposition/incorporation of P**. The P incorporation may be electrochemically driven or chemically assisted. It may involve adsorption of phosphite/hypophosphite species on the growing metal surface, followed by electron transfer and incorporation as P or phosphide species. The local overpotential, mass transport (diffusion), and the ratio of P precursor to Co concentration influence how much P is incorporated. Under conditions favoring high P incorporation, the deposit tends toward amorphous structure (high P helps suppress LRO). **(3) Suppression of crystallinity**. High P content disrupts regular lattice formation, leading to a disordered (amorphous) structure. The presence of P in interstitial or substitutional sites disrupts metallic Co crystallization. Also, the deposition conditions (e.g., high overpotential, fast deposition, limited diffusion, pulsed/reverse plating) can kinetically “freeze” the growth in a non-equilibrium (amorphous) state. Indeed, pulsed-reverse plating has been used to obtain amorphous CoP films. The surface morphology evolution during electrodeposition suggests that early stages favor fine granular/amorphous deposition which later may coarsen or develop partial crystallinity if P content is low. **(4) Growth and morphological evolution**. As deposition continues, the film thickens; diffusion of ionic species, changes in local pH, depletion zones, and local current density heterogeneities may lead to microstructural variation. Roughness, nonuniform current density, and local variations in P precursor concentration can lead to mixed-phase regions (amorphous + nanocrystalline) or loss of purely amorphous character, especially when the P incorporation drops. For example, if the localized current density is too high, crystallites may begin to nucleate, undermining full amorphous structure. **(5) Post-treatment/stability**. The as-deposited films are often stable in the amorphous form at room temperature, but upon annealing or high-temperature exposure, crystallization may occur (leading to Co and Co–P crystalline phases). Thus, the electrodeposited CoP film’s structure is a result of the competition between deposition kinetics (favoring rapid, disordered growth) vs. atomic rearrangement (which favors ordering). A high P content, moderate overpotential, and careful control of deposition parameters are key to stabilizing the amorphous phase.

**Figure 8:**
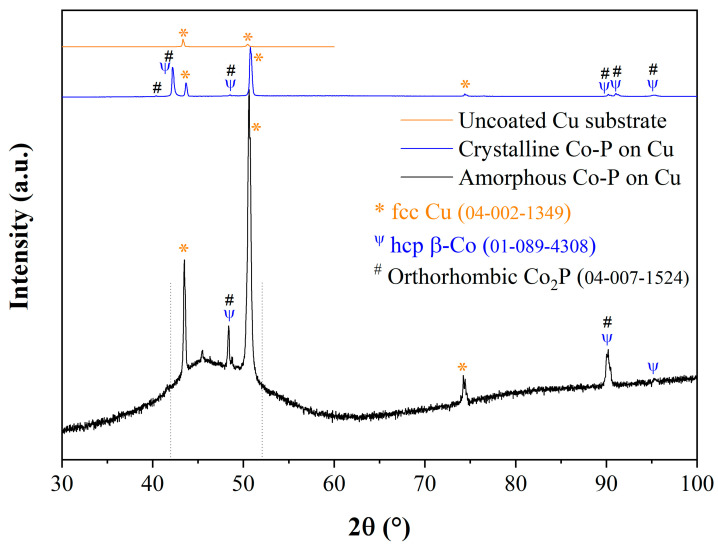
RT-XRD of amorphous and crystalline CoP coatings on Cu, deposited from solution No. 1. The region corresponding to the amorphous halo is marked by the two vertical dotted lines.

Both amorphous (i.e., from stirred solutions) and crystalline (i.e., from unstirred solutions) coatings on Cu substrates were characterized by RT-XRD. Figure 8 shows the diffractograms from the uncoated Cu substrate, a sample coated for 1.2 h with amorphous CoP from solution No. 1, and a sample coated for 1.2 h with crystalline CoP from solution No. 1. In both coated samples, there were three reflections at ca. 43°, 50°, and 74°, which are related to the (111), (200), and (220) reflections from the fcc Cu substrate (ICDD PDF-4+ 04-002-1349), respectively [34]. The first two reflections appeared in the diffractogram of uncoated Cu, which extended only to 2θ = 60°. In samples coated for 1.2 h with “amorphous” CoP from solution No. 1 (as well as from solution Nos. 0 and 2, not shown herein), the X-ray diffractograms contained some weak reflections from hexagonal β-Co (PDF 01-089-4308, space group *P*6_3_/*mmc* (194)), (101) at ca. 48.4°, and (200) at ca. 90.2°. These two reflections also appeared in the diffractogram of the crystalline CoP sample, which included a strong (100) reflection at ca. 42.2° and a (201) reflection at ca. 95.2°, also from the hcp Co phase. The presence of these reflections indicates the coexistence of nanocrystalline Co. One cannot conclude based on the XRD results alone that the nanocrystalline phase is actually orthorhombic Co_2_P (ICDD PDF-4+ 04-007-1524, space group *Pnma* (62)) with the observed reflections at 40.4°, 42.2°, 48.5°, 90.2°, 90.5°, 91.1°, and 95.3° presumably corresponding to its (112), (202), (013), (421), (132), (404), and (422) planes, respectively. Based on the XRD data alone, the formation of a cubic Cu_0.5_Co_0.5_ intermetallic compound (PDF 04-020-2827 space group $\mathrm{Fm}\bar{3}m$ (225)) cannot be ruled out either; the formation of this phase could explain the strong adhesion between the substrate and the coating [74]. It should be noted that electron diffraction supported the existence of hcp β-Co and ruled out the existence of orthorhombic Co_2_P (see below). The diffractogram from the coating deposited for 1.2 h from solution No. 7 (not shown herein) included the primitive cubic ε-Co polymorph (PDF 04-017-5578, space group *P*4_1_32 (213)), a crystal structure of Co nanoparticles that is well represented in the literature [77,78]. It is a non-compact, metastable cubic crystal structure that can be transformed upon annealing into the more stable hcp and fcc structures.

The background in the diffractogram of uncoated Cu was flatter than that of the coated samples, indicating the presence of an amorphous phase in the coated samples. A superimposed amorphous halo was evident between ca. 42° and 52° in the diffractograms of the coated samples. In previous publications, an amorphous halo (or a broad peak related to a nanocrystalline phase, possibly in combination with an amorphous phase) was observed between 40° and 50° [59], for example at 43° [47]. The reflection at ca. 25° is related to the sample holder.

**Figure 9:**
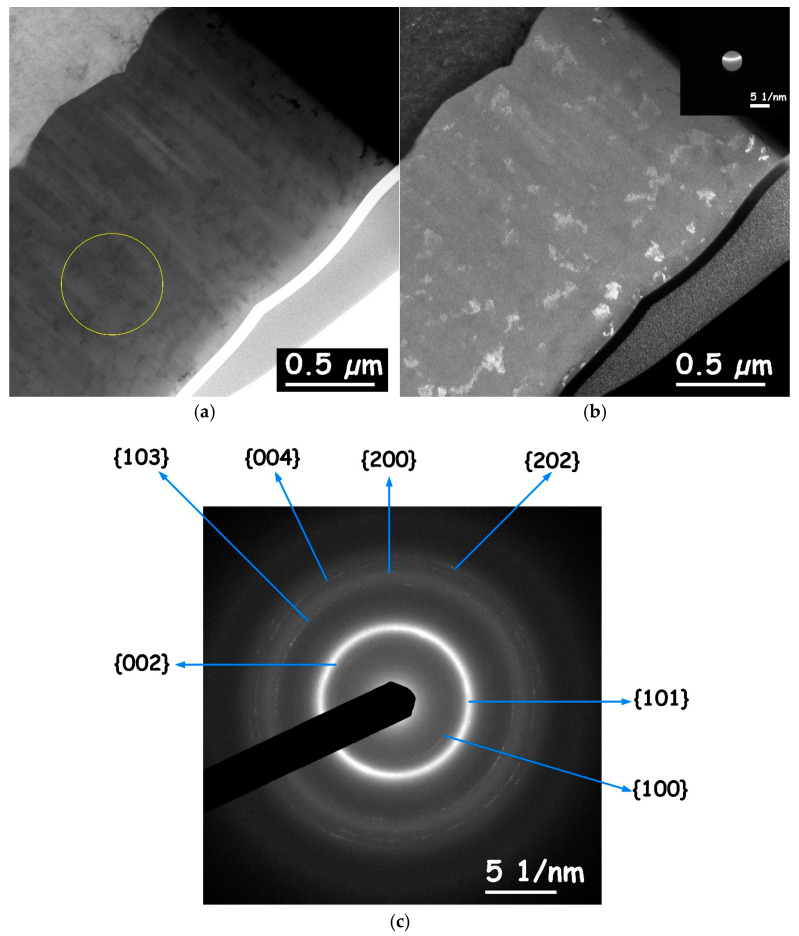
BF TEM micrograph (**a**) and DF TEM micrograph (**b**). Electron microdiffraction pattern (**c**) of CoP coating deposited for 1.2 h from a stirred solution No. 1. The yellow circle in the BF TEM micrograph marks the region from which the microdiffraction (parallel illumination) was collected. The DF micrograph was constructed from a region of the scattering pattern of around ~0.5 Å^−1^, which is attributed to scattering of Co phases (both hcp and fcc), see inset. The reflection rings in the microdiffraction are indexed as hcp Co using the three-index system.

TEM samples were prepared from the nominal amorphous CoP coatings (solution No. 1, with stirring) by FIB. One sample was first electrodeposited from stirred solution No. 1 for 1.2 h, whereas the second sample was electrodeposited for 4 h. Figure 9 shows BF and DF TEM images as well as an electron microdiffraction pattern (parallel illumination) from the sample coated for 1.2 h. The diffraction pattern shows diffuse (halo) rings of elastic electron scattering, which are indicative of amorphous solids due to short-range order [79]. The coexistence of nanoscale crystallites is evident from reflection spots observed in the scattering pattern, as well as from the diffraction contrast in the images. The mostly uniform radial scattering of electrons is related to the lack of crystallographic texture. The surface morphology from SEM imaging and X-ray diffractograms both indicated the presence of nanocrystallites. It can therefore be assumed that the nanocrystallites in Figure 9 were not artifacts from FIB sample preparation.

Inoue suggested [80] an empirical rule according to which the difference in the atomic radii of the main elements should be above ca. 12% in order to trigger the formation of the amorphous phase. For the CoP system, this difference is 15.3%. With the increase of P concentration, P atoms will inhibit nucleation and growth of the Co-based crystalline phase, and above a critical concentration the structure will become completely amorphous [74].

**Figure 10:**
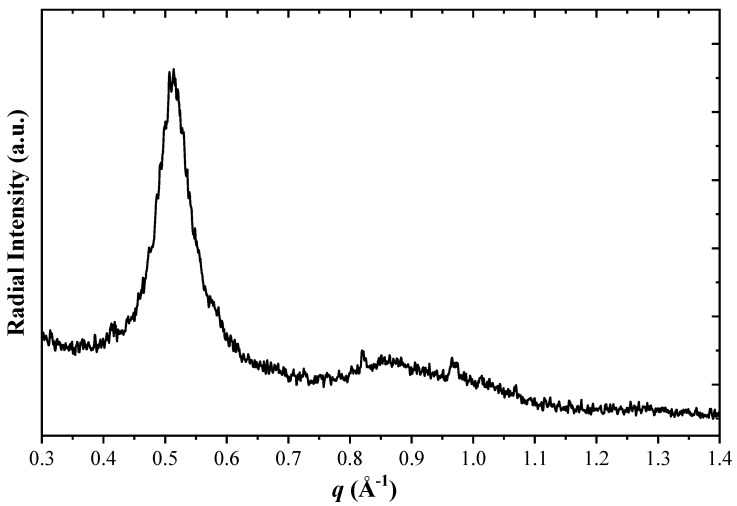
The measured radial intensity as a function of scattering vector size, ***q***, extracted from a parallel illumination microdiffraction of a CoP electrodeposit.

To further identify crystalline phases, one can extract the radial intensity of the electron diffraction as a function of the scattering vector. For example, see the procedure in Kohn et al. [81] for electroless deposited Co_0.9_W_0.02_P_0.08_ films before and after thermal annealing. Comparing that report to the current study (Figure 10), it can be concluded that the nanoscale crystalline phase in the CoP electrodeposited alloy is most likely the hcp phase Co due to the lack of a clear fcc Co(200) reflection at *q* = 0.564 Å^−1^. Therefore, the Co phase is identified as hcp, not the fcc phase. Furthermore, orthorhombic Co_2_P cannot be detected due to a lack of reflection intensities around 0.6 and 0.8 Å^−1^, thus clarifying the uncertainty in indexing of XRD reflections in Figure 8. The peak at approximately *q* = 0.514 Å^−1^ represents interplanar crystallographic distances of ca. 2 Å attributed to (101) and (002) reflections of hcp Co.

**Figure 11:**
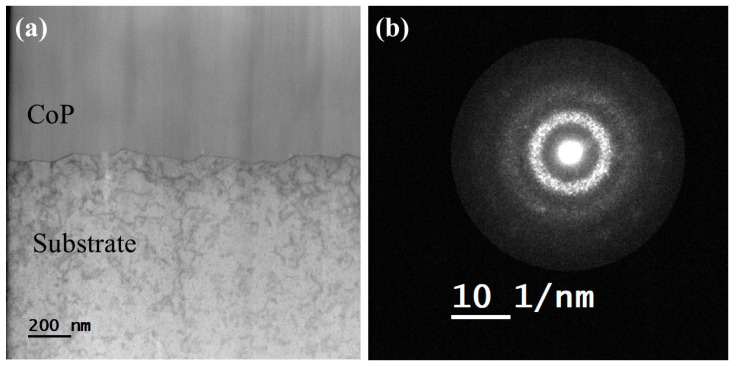
(**a**) Cross-sectional BF STEM image from the CoP coating. (**b**) Electron diffraction obtained in STEM mode from the CoP coating indicating the presence of nanoscale crystals (reflection spots).

In an attempt to suggest a microstructural explanation of the exceptionally strong adhesion between the CoP coating and the Cu substrate, the interface between the substrate and the coating was studied using an aberration-corrected STEM. Figure 11a shows a BF STEM image (angular range of the detector up to 64 mrad) from a 1.2 μm thick CoP coating. The contrast of the BF STEM image for the CoP coating appears mostly homogenous, with a lack of obvious diffraction contrast. The streaks are attributed to curtaining effects during the preparation of the sample in the FIB. Figure 11b shows an electron diffraction pattern obtained in STEM mode from this region. Here, both halo rings characteristic of an amorphous phase and spots characteristic of nanoscale crystals are apparent.

**Figure 12:**
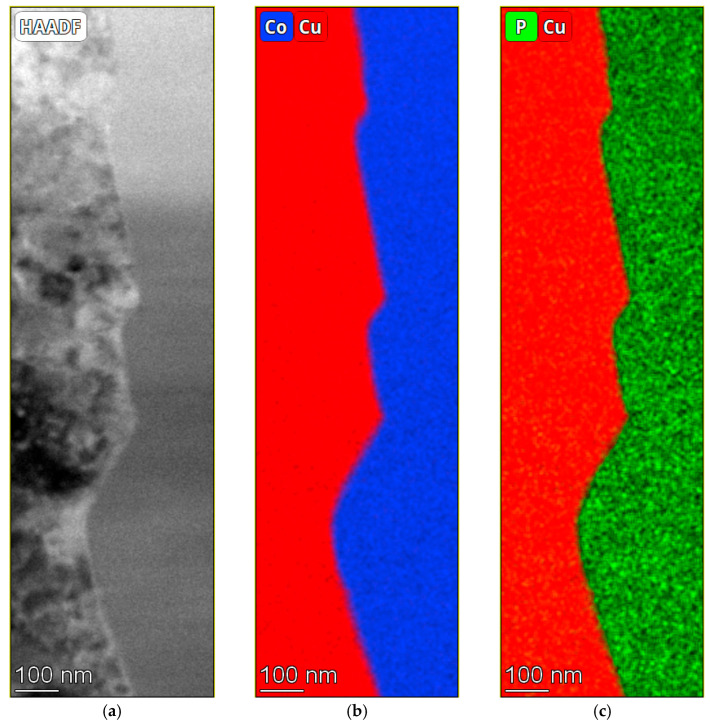

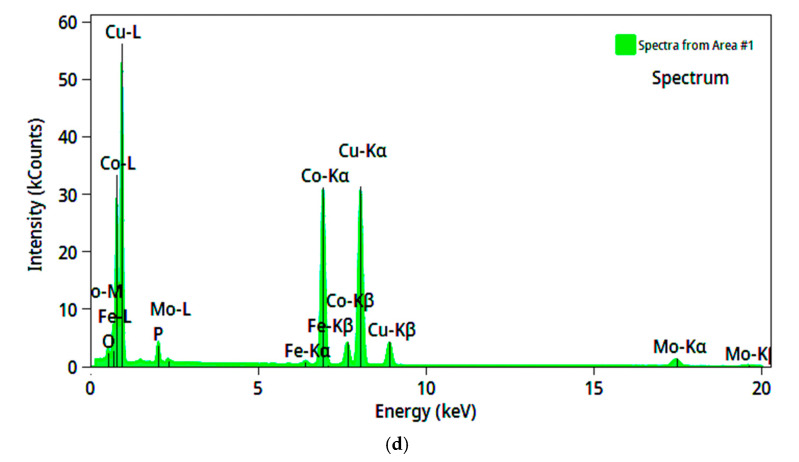
Top: HAADF STEM image (**a**), elemental mapping based on Cu-K/Co-K (**b**) and Cu-K/P-K (**c**) characteristic x-ray photons, as extracted from STEM EDS measurements, around the interface between the Cu substrate and the CoP coating. (**d**) A typical example of an EDS spectrum collected by STEM from a region that contains both the substrate and coating.

A high-angle annular dark field (HAADF) STEM image, along with Cu/Co and Cu/P elemental mapping by EDS around the interface between the Cu substrate and a 1.2 μm thick CoP coating, are shown in Figure 12a–c. The characteristic X-ray photon energies that were used for the EDS mapping were the K-shell for Cu (*K*_α1_ = 8.0478 keV, *K*_α2_ = 8.0279 keV, *K*_β_ = 8.9055 keV), the K-shell for Co (*K*_α1_ = 6.903 keV, *K*_α2_ = 6.9150 keV, *K*_β1_ = 7.6494 keV), and K-shell for P (*K*_α1_ = 2.0133 keV). It is evident that the interface is wavy (rough), and that the Co and P elemental maps match one another in distribution, implying that the coating is an alloy of Co with P. The wavy interface makes it difficult to determine from these STEM measurements whether there is chemical intermixing and the formation of intermetallic compounds at the interface, which could explain the exceptionally strong adhesion. Figure 12d shows a typical EDS spectrum acquired from a region including both the coating and the substrate during STEM measurements. Characteristic energy peaks are detected of Co and P from the coating, Cu from the substrate, Fe from the objective pole piece of the TEM, and Mo from the grids used during the FIB preparation, are evident.

**Figure 13:**
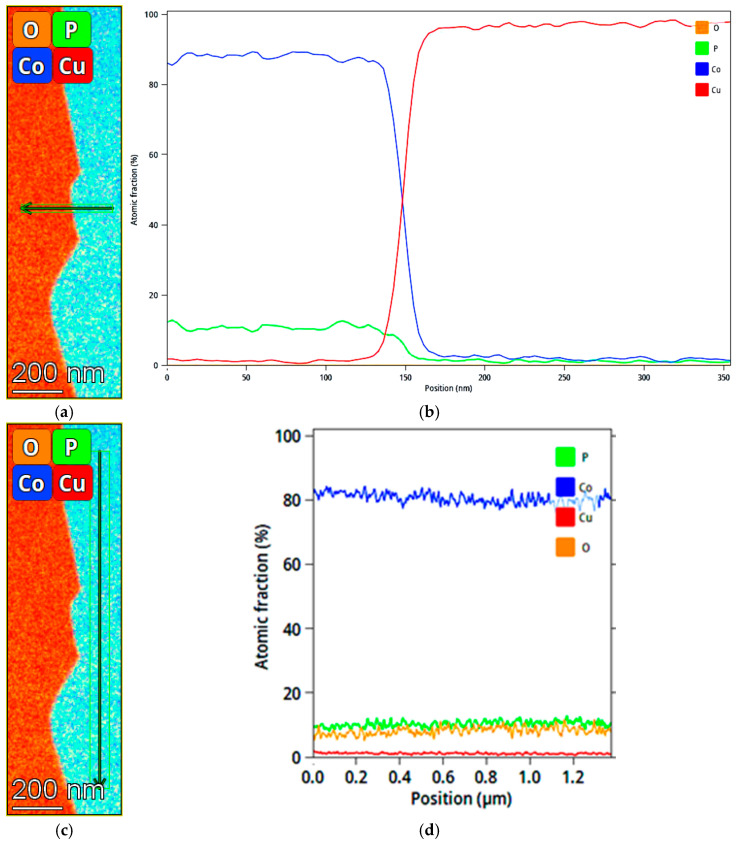
STEM-EDS quantified elemental line scan across the substrate/coating interface extracted from the elemental mapping (**a**,**b**) and within the coating (**c**,**d**).

Figure 13 shows a line scan of elements, quantified by EDS mapping across the interface between the substrate and coating as well as within the coating itself. No indication exists for a significant chemical reaction between the Co-based coating and the Cu substrate. It should be noted that the surface roughness of the Cu substrate was approximately 46 nm, i.e., large relative to the TEM sample thickness. Fitting the width of the signal to an error function between fractions of 0.84 and 0.16 [82] of the maximal Co content at the interface area results in a sigma value of approximately 16 nm, comparable to the surface roughness. Thus, we conclude that the measurements follow this roughness, so we cannot detect possible interaction between the Cu substrate and CoP coating. Furthermore, support for the effect of surface roughness on strong adhesion rather than intermixing is that the diffusion coefficient for Co in Cu at RT is relatively small (1.16 × 10^−38^ cm^2^/s by calculation [83]).

The chemical composition within the coating is uniform, and the nearly constant concentration of oxygen on both sides of the interface implies that there was some oxidation of the whole sample (e.g., during storage) rather than formation of a CoO phase during electrodeposition. Oxidation could also be on the surfaces of the TEM lamella. The chemical composition on the substrate side was 99.06 (±20.00) wt.% Cu and 0.94 (±0.18) wt.% Co. The chemical composition on the coating side was 93.72 (±17.08) wt.% Co, 5.61 (±0.70) wt.% P, and 0.66 (±0.13) wt.% Cu. These STEM-EDS based measurements match nicely the results of SEM-EDS (93.38 wt.% Co, 6.62 wt.% P).

### 3.7. Thermal Stability of the CoP Coatings

DSC tests were carried out on pure Cu, presumably amorphous CoP on Cu, and presumably crystalline CoP on Cu. CoP was analyzed in the form of a coating (and not in powder form) because it was impossible to separate the coating from the substrate mechanically, chemically (in H_2_O_2_ + HCl solution), or with a laser (i.e., evaporation by heat using a 532 nm laser).

**Figure 14:**
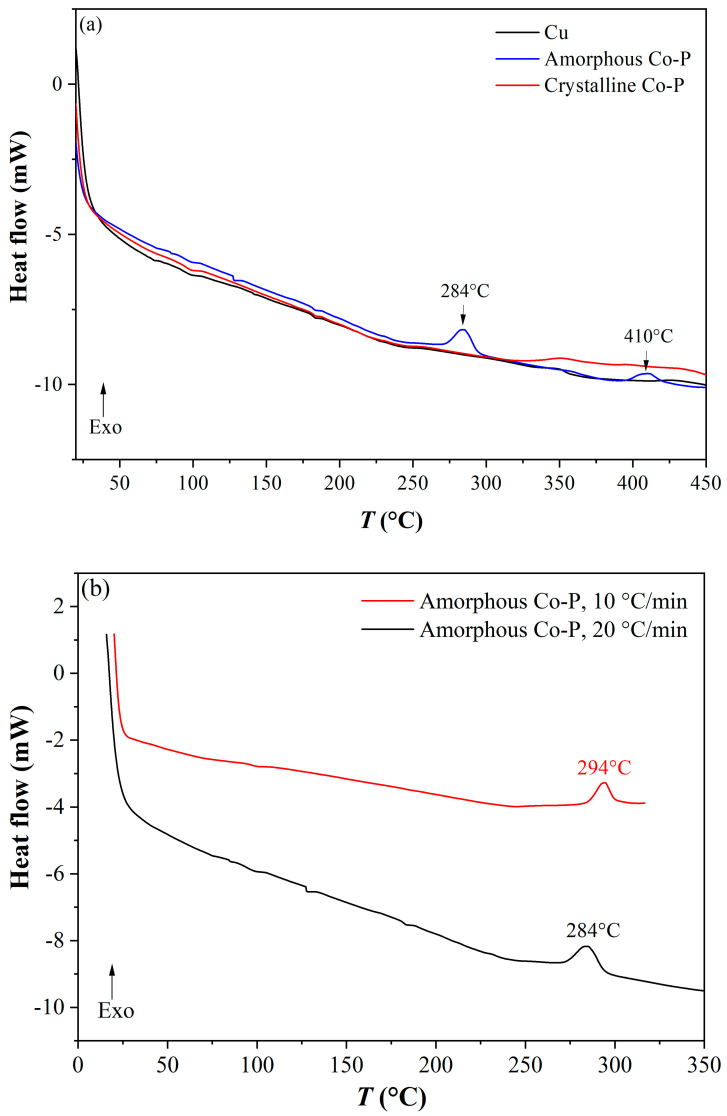

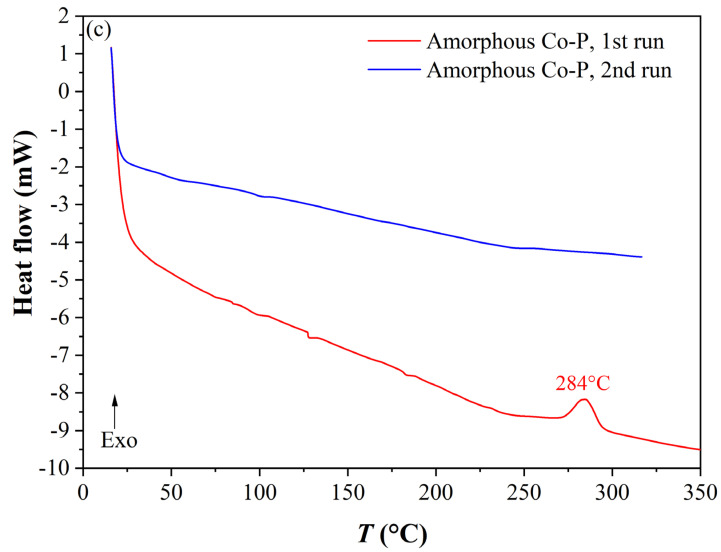
DSC spectra. (**a**) Pure Cu, Cu coated with crystalline CoP, and Cu coated with amorphous CoP. Heating rate: 20 °C/min. (**b**) Cu coated with amorphous CoP. Heating rates: 20 and 10 °C/min. (**c**) First and second DSC runs of an initially amorphous CoP coating on Cu. Heating rate: 10 °C/min.

DSC curves of the three samples are shown in Figure 14a using a heating rate of 20 °C/min. For pure Cu and Cu coated with a crystalline CoP film, neither exothermic nor endothermic heat flow peaks were evident. This supports the SEM and XRD observations that the CoP coating deposited from the unstirred solution was crystalline. In contrast, the sample coated from a stirred solution showed a broad (“mountain”) exothermic peak at ca. 284 °C. A second shallower exothermic peak is evident in the DSC curve of the sample coated with a stirred solution at ca. 410 °C. Amorphous CoP coatings on Cu were DSC-tested also at a lower heating rate of 10 °C/min (see Figure 14b). The exothermic peak in this case was at 294 °C. When the heating rate was higher, the heat flow was higher too. Because the samples contained Cu in addition to CoP, it was not possible to make any enthalpy measurements. Finally, the amorphous CoP-coated sample that was tested at 10 °C/min was cooled down to RT and then heated again. As evident from Figure 14c in the second run, the first exothermic peak did not appear again, implying that the amorphous phase in the parent coating fully transformed to a crystalline phase during the first run (an irreversible process).

Crystallization of the amorphous and hybrid (amorphous + crystalline) CoP electrodeposits through two (or more) consecutive steps has been reported before based on non-isothermal DSC tests and complementary microstructural characterization. Cebollada et al. [84] studied Co_100-*x*_P*_x_* (10 < *x* < 25 at.%) electrodeposits and observed two-step crystallization for *x* < 21 at.%. For *x* = 10.5 at.%, which is most similar to the composition of the layers from solution No. 1 in the present work, two broad peaks that were not fully resolvable were observed (Figure 1 in Ref. [84]), one at ca. 287 °C and the other at ca. 387 °C (also at a heating rate of 10 °C/min). These temperature values are similar to those observed in the current work. The peak at the higher temperature was related to a polymorphic (solid-state) phase transformation from α-Co to β-Co. The final product in that case was a mixture of crystalline Co and Co_2_P. Masui et al. [85] studied the crystallization kinetics of amorphous Co_84.8_P_15.2_ and Co_81.6_P_18.4_ (at.%) electrodeposits. It was concluded that in CoP alloys containing between ca. 5 and 9 wt.% P (the range where the compositions in the current research fall), hcp-Co separates in the amorphous matrix at 277–337 °C, and upon further heating the matrix transforms into stable phases of hcp-Co and Co_2_P by eutectic crystallization at 337–397 °C. Vijayan et al. [86] studied the crystallization of crystalline (<10.0 at.% P), hybrid crystalline/amorphous (10.0–12.1 at.% P), and amorphous (>12.1 at.% P) coatings. Differentiation was made between hypoeutectic and hypereutectic amorphous CoP (the eutectic composition being 19.9 at.% P, see the CoP equilibrium binary phase diagram in Ref. [87]). It was hypothesized that the two peaks in the DSC curves of initially fully amorphous hypoeutectic alloys corresponded to short-range atomic movements of P-atoms surrounding Co-rich clusters and longer-range diffusion resulting in the formation of fcc-Co and Co_2_P, respectively.

**Figure 15:**
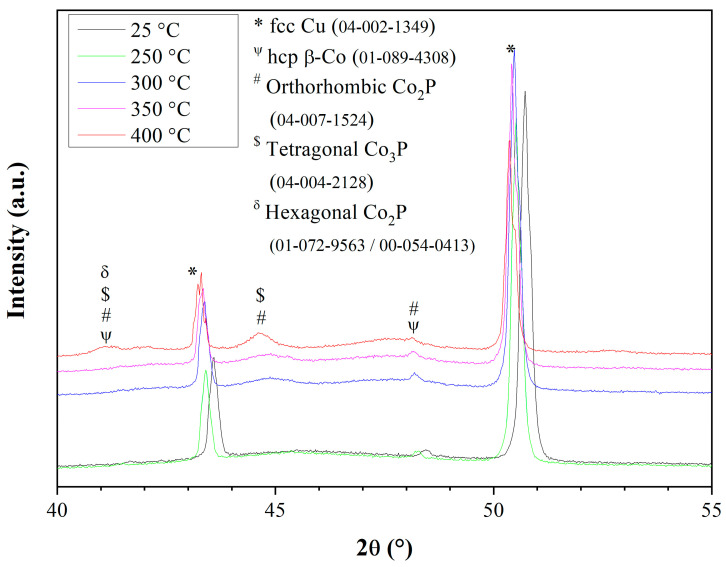
High-temperature XRD of CoP electrodeposits from stirred solution No. 1.

The temperature-dependent phase transformations were also studied by HT-XRD. These runs were conducted at 25, 100, 150, 200, 250, 300, 350, and 400 °C in a nitrogen (N_2_) environment at a heating rate of 10 °C/min. Selected results are shown in Figure 15, which should be compared to Figure 8. The (111) and (200) reflections from the fcc Cu substrate (ICDD 04-002-1349) appear in Figure 15 as well. The (101) reflection from hcp β-Co (PDF 01-089-4308) appears at ca. 48.4°. This reflection could also be ascribed to the (013) reflection of the orthorhombic Co_2_P phase (ICDD 04-007-1524), but an examination of electron scattering patterns removed this possibility. As the temperature was raised, a broad peak of a Co*_x_*P*_y_* phase evolved at ca. 44.6°. This reflection may be ascribed to the (130) reflection of orthorhombic Co_2_P or to the (112) reflection of the tetragonal Co_3_P phase (PDF 04-004-2128, space group $I\bar{4}$ (82)). Another broad peak evolved at 400 °C at ca. 41.1°. It could be related to the (100) plane of hcp β-Co (PDF 01-089-4308), the (201) reflection of orthorhombic Co_2_P (PDF 00-032-0306), the (210) reflection of the orthorhombic Co_2_P phase (ICDD 04-007-1524), the (400) reflection of the tetragonal Co_3_P phase (PDF 04-004-2128), or the (111) plane of hexagonal Co_2_P (PDF 01-072-9563 or PDF 00-054-0413, space group $P\bar{6}2m$ (189)). It may thus be concluded from HT-XRD that crystallization occurs above 250 °C, in accordance with the DSC data. However, different stoichiometries and symmetries may coexist together, making the analysis unambiguous. It is evident from Figure 15 that as the temperature was raised, the peaks were shifted toward lower angles.

Finally, what could explain the observation that stirring the solution resulted in amorphous coatings, whereas leaving the solution unstirred led to crystalline coatings? The structural transition observed between amorphous and crystalline CoP coatings under different hydrodynamic conditions can be rationalized by considering the influence of mass transport and local interfacial chemistry during electrodeposition. In the stirred CoSO_4_·7H_2_O–H_3_PO_3_ bath, enhanced convection promotes rapid renewal of electrolyte species at the cathode, reducing the thickness of the diffusion layer and removing hydrogen bubbles more effectively. This maintains a **lower local pH** at the electrode surface and facilitates the electrochemical reduction of H_3_PO_3_, leading to **greater phosphorus incorporation** into the deposit. High P contents are well known to suppress LRO and yield **amorphous Co–P coatings** [4,5,6,22]. In contrast, under unstirred (quiescent) conditions, limited mass transport allows hydrogen accumulation and a pH rise near the surface, which decreases the rate of P codeposition. The resulting lower P content favors **Co-rich, crystalline Co–P phases** with distinct diffraction peaks corresponding to orthorhombic CoP or Co_2_P structures. Additionally, vigorous stirring enhances the instantaneous nucleation rate, producing fine-grained, disordered films, while quiescent deposition is more diffusion-limited, enabling larger grain growth and crystallization.

## 4. Conclusions

Here, CoP amorphous coatings were electrodeposited on a Cu substrate from a stirred solution that contained cobalt (II) sulfate, glycine, and phosphorous acid in deionized water. The pH of the solution was about 3.7. A crystalline CoP coating was also electrodeposited from an unstirred solution for comparison. The electrodeposition of both amorphous and crystalline CoP coatings was performed in an air atmosphere at room temperature. The best coating consisted of ca. 93.6 wt.% Co and 6.4 wt.% P. An outstanding adhesion of the CoP amorphous coating to the substrate was observed. The coating had excellent mechanical properties—a low coefficient of friction (0.14 ± 0.02) and a high hardness (7.8 ± 0.7 GPa). The contour of the coating followed the substrate surface and its asperities. This could explain the strong adhesion of the CoP coating via mechanical interlocking. The microstructure was uniform and hybrid (i.e., nanocrystals embedded in an amorphous matrix). The coating had good thermal stability and exhibited a two-step crystallization at 284 °C and between 300 and 400 °C.

## Figures and Tables

**Table 1:** Six different solutions for electrodeposition of CoP.

Solution No.	CoSO_4_·7H_2_O [M]	H_3_PO_4_ [M]	H_3_PO_3_ [M]	Glycine [M]	pH
0	0.25	0.102	0.122	0.3	3.6–3.8
1	0.25	-	0.122	0.3	3.72
2	0.25	-	0.122	0.6	3.83
3	0.25	-	0.122	0.6	9.71
4	0.10	-	0.122	0.6	9.71
7	0.10	-	0.122	0.6	6.19

**Table 2:** *R*a values measured by CSLM on the three surfaces.

Sample	*R*a [µm]
Uncoated Cu	0.049 ± 0.002
Amorphous CoP coating on Cu substrate	0.046 ± 0.001
Crystalline CoP coating on Cu substrate	0.052 ± 0.001

**Table 3:** Typical chemical compositions (based on EDS) of CoP coatings deposited from different solutions for different deposition times, with or without stirring. *n* is the number of spectra acquired at different locations.

Solution No.	Stirring	*t* [h]	Co [wt.%]	P [wt.%]	Co [at.%]	P [at.%]
0	Y	1.2	94.83 ± 1.34 (*n* = 4)	5.17 ± 1.34 (*n* = 4)	90.62 ± 2.33 (*n* = 4)	9.38 ± 2.33 (*n* = 4)
1	Y	1.2	93.61 ± 0.46 (*n* = 16)	6.39 ± 0.46 (*n* = 16)	88.50 ± 0.77 (*n* = 16)	11.50 ± 0.77 (*n* = 16)
1	N	1.2	93.22 ± 1.76 (*n* = 2)	2.66 ^‡^ ± 0.20 (*n* = 2)		
1	Y	4	92.80 ± 0.65 (*n* = 29)	7.20 ± 0.65 (*n* = 29)	87.14 ± 1.09 (*n* = 29)	12.86 ± 1.09 (*n* = 29)
1	N	4	94.53 (*n* = 1)	5.47 (*n* = 1) 2.33 ^§^	90.09 (*n* = 1)	9.91 (*n* = 1)
2	Y	1.2	93.33 ± 1.22 (*n* = 11)	6.67 ± 1.22 (*n* = 11)	88.05 ± 2.06 (*n* = 11)	11.95 ± 2.06 (*n* = 11)
7	Y	1.2	98.30 ± 0.53 (*n* = 2)	1.70 ± 0.53 (*n* = 2)	96.80 ± 0.97 (*n* = 2)	3.20 ± 0.97 (*n* = 2)

^§^ 3.03 wt.% O was detected by EDS in this coating, ^‡^ 3.00 wt.% O and 1.12 wt.% C were detected by EDS in this coating.

**Table 4:** Coefficient of dry friction of amorphous CoP coating on Cu substrate. For each sample, the average and standard deviation of results from at least 3 test coupons are given.

Sample Number	Coefficient of Friction	P [wt.%]
4.3.1 ^§^	0.11 ± 0.02	7.19 ± 0.46
4.3.2 ^§^	0.14 ± 0.02	6.55 ± 0.36
4.3.3 ^§^	0.12 ± 0.01	6.91 ± 0.72
4.3.4 ^§^	0.15 ± 0.02	6.13 ± 0.42
3.6.2	0.16 ± 0.01	6.58 ± 0.17
3.5.1	0.17 ± 0.01	6.84 ± 0.07
3.6.1	0.14 ± 0.01	6.73 ± 0.23
3.3.2	0.12 ± 0.01	9.83 ± 0.65

^§^ Samples 4.3.1 through 4.3.4 were electrodeposited simultaneously in different cells under identical conditions, using a multichannel potentiostat/galvanostat—samples No. 4.3.1 and 4.3.3 in one channel, samples No. 4.3.2 and 4.3.4 in a second channel. Disturbance of the CE occurred shortly during deposition of sample No. 4.3.4.

**Table 5:** Chemical composition of the amorphous and crystalline CoP coatings in samples for nanoindentation tests.

Microstructure	P		Co	
	[wt.%]	[at.%]	[wt.%]	[at.%]
Amorphous	7.83 ± 0.21	13.91 ± 0.35	92.17 ± 0.21	86.99 ± 0.35
Crystalline	4.30 ± 0.17	7.88 ± 0.30	95.70 ± 2.17	92.12 ± 0.30

**Table 6:** Elastic modulus and hardness of amorphous and crystalline CoP coatings based on nanoindentation measurements.

Microstructure	*E* [GPa]	*H* [GPa]	*H* [VHN] ^‡^
Amorphous	153 ± 9 ^§^	7.8 ± 0.7	795.6
Crystalline	167 ± 17	4.9 ± 0.8	449.8

^§^ std. error of the mean values. ^‡^ H [VHN] by converting units from GPa (which is expressed in N/m^2^) to kg_f_/mm^2^ (ratio of 0.009807).

## Data Availability

The original contributions presented in this study are included in the article. Further inquiries can be directed to the corresponding author.
